# *Eimeria bovis* Macromeront Formation Induces Glycolytic Responses and Mitochondrial Changes in Primary Host Endothelial Cells

**DOI:** 10.3389/fcimb.2021.703413

**Published:** 2021-07-15

**Authors:** Zahady D. Velásquez, Sara López-Osorio, Sybille Mazurek, Carlos Hermosilla, Anja Taubert

**Affiliations:** ^1^ Institute of Parasitology, Biomedical Research Center Seltersberg, Justus Liebig University of Giessen, Giessen, Germany; ^2^ Research Group CIBAV, School of Veterinary Medicine, Faculty of Agrarian Sciences, University of Antioquia, Medellin, Colombia; ^3^ Institute of Veterinary Physiology and Biochemistry, Justus Liebig University of Giessen, Giessen, Germany

**Keywords:** apicomplexan parasites, *Eimeria bovis*, endothelial cell, senescence, mitochondrial damage, reactive oxygen products

## Abstract

*Eimeria bovis* is an intracellular apicomplexan parasite that causes considerable economic losses in the cattle industry worldwide. During the first merogony, *E. bovis* forms large macromeronts with >140,000 merozoites I in host endothelial cells. Because this is a high-energy demanding process, *E. bovis* exploits the host cellular metabolism to fulfill its metabolic requirements. We here analyzed the carbohydrate-related energetic metabolism of *E. bovis*–infected primary bovine umbilical vein endothelial cells during first merogony and showed that during the infection, *E. bovis*–infected culture presented considerable changes in metabolic signatures, glycolytic, and mitochondrial responses. Thus, an increase in both oxygen consumption rates (OCR) and extracellular acidification rates (ECAR) were found in *E. bovis*–infected host cells indicating a shift from quiescent to energetic cell status. Enhanced levels of glucose and pyruvate consumption in addition to increased lactate production, suggesting an important role of glycolysis in *E. bovis*–infected culture from 12 days p.i. onward. This was also tested by glycolytic inhibitors (2-DG) treatment, which reduced the macromeront development and diminished merozoite I production. As an interesting finding, we observed that 2-DG treatment boosted sporozoite egress. Referring to mitochondrial activities, intracellular ROS production was increased toward the end of merogony, and mitochondrial potential was enhanced from 12 d p. i. onward in *E. bovis*–infected culture. Besides, morphological alterations of membrane potential signals also indicated mitochondrial dysfunction in macromeront-carrying host endothelial culture.

## Introduction

*Eimeria bovis* is an obligate intracellular parasite that belongs to the subphylum Apicomplexa. It represents one of the most pathogenic *Eimeria* species in cattle coccidiosis, causing severe hemorrhagic typhlocolitis in calves (Daugschies and Najdrowski, 2005). *E. bovis* typically replicates within lymphatic host endothelial host cells of the lacteals of the ileum villi during first merogony (Hermosilla et al., 2012). Mature macromeronts contain thousands of merozoites (>140,000 merozoites I) that give rise to intracellular structures of up to 300 µm in size *in vivo* (Nyberg and Hammond, 1965). *In vitro*, *E. bovis* macromeront development takes approximately 24 days until merozoite type I production takes place (Hermosilla et al., 2002) and increased demand of energy and cell building blocks for phospholipids, cholesterol, nucleic acids, amino acids, proteins, and organelles (Taubert et al., 2010; Hermosilla et al., 2012). Because of its restricted metabolic capacities, the parasite needs to modulate the host cellular metabolism to supply infected host cells with energetic compounds. In addition to oxidative phosphorylation (OXPHOS), the carbohydrate catabolism, such as glycolysis and tricarboxylic acid (TCA cycle), is the most important pathway to produce energy *via* ATP. Former transcriptomic analyses of *E. bovis*–infected host endothelial cells indicated an impact of this obligate intracellular parasite on host cellular carbohydrate, amino acid, and folic acid metabolism, as well as energy production because several enzymes, such as galactose-1-phosphate uridylyltransferase, UDP-glucose glycoprotein glucosyltransferase 2, methylene-tetrahydrofolate-dehydrogenase, malic enzyme 1, or malate dehydrogenase 1, were found significantly up-regulated at 14 days post infection (p. i.) (Taubert et al., 2010). Overall, a sub-set of up-regulated genes in *E. bovis*–infected host cells revealed to be involved in carbohydrate synthesis and metabolism, such as amino sugar, fructose/mannose, galactose, nucleotide sugar, and pyruvate metabolism, as well as in glycolysis/gluconeogenesis and the pentose phosphate pathway (Taubert et al., 2010). Although very little is known on *E. bovis*-own metabolic capacities, much more details on metabolic equipment and metabolic dependencies on the host cell have been reported for other apicomplexan parasites. *Toxoplasma gondii* and *Plasmodium* spp. own the complete set of proteins related to glycolytic pathway, TCA cycle enzymes, and mitochondrial electron transport chain (Ralph, 2005; Fleige et al., 2007; Polonais and Soldati‐Favre, 2010). The lytic cycle of *T. gondii* was demonstrated to rely on glucose and glutamine for macromolecule biosynthesis (ATP, nucleic acids, proteins, and lipids) (Nitzsche et al., 2016). However, the amount of energy regenerated *via* glucose and glutamine degradation by *T. gondii* alone seems insufficient to maintain parasite survival and virulence, hence it must be supplemented by host cell metabolism (Crawford et al., 2006; Blume et al., 2009; MacRae et al., 2012; Oppenheim et al., 2014; Blume et al., 2015; Tymoshenko et al., 2015; Nitzsche et al., 2016). For fatty acid synthesis, *T. gondii* and *P. falciparum* rely on the prokaryotic type II fatty acid synthesis pathway (FASII) (Waller et al., 1998). For instance, *P. falciparum* uses FASII during the asexual blood and late liver stages (Vaughan et al., 2009), whereas *T. gondii* uses FASII during tachyzoite development, apicoplast biogenesis, and parasite proliferation (Mazumdar et al., 2006; Ramakrishnan et al., 2015). Apicomplexan *T. gondii, Cryptosporidium parvum, Besnoitia besnoiti*, and *E. bovis* all scavenge nutrients of host cells, such as glucose and glutamine, which serve as energy sources, as well as precursors for cholesterol and purine/pyrimidine synthesis (Striepen et al., 2004; Taubert et al., 2010; Ehrenman et al., 2013; Coppens, 2014; Hamid et al., 2015; Taubert et al., 2016). In *Theileria* infections, lymphocyte transformation is characterized by a shift from mitochondrial oxidative phosphorylation to cytosolic lactate production (Medjkane et al., 2014), a very characteristic metabolic feature of tumor cells that are well known as the Warburg effect (Medjkane and Weitzman, 2013; Medjkane et al., 2014; Metheni et al., 2015). In contrast to other apicomplexan parasites, *C. parvum* entirely lacks enzymes necessary for TCA cycle, cytochrome-based respiratory chain, and *de novo* biosynthesis of fatty acids, nucleotides, and amino acids. All these findings strongly evidence *C. parvum* dependence on host cell metabolism to achieve its replication (Abrahamsen et al., 2004; Zhu, 2004; Vélez et al, 2021), thereby giving novel insights into a wide array of adaptative strategies used by apicomplexans related to their modulation capacities of host cell metabolism.

Former studies showed that *Eimeria* species effectively modulate several functions of the host cell. Thus, *E. bovis* alters cytoskeleton dynamics (Hermosilla et al., 2008), apoptosis (Lang et al., 2009), immunomodulatory molecule production (Hermosilla et al., 2006), host cellular cell cycle (Vélez et al., 2021), and host cellular metabolism (Taubert et al., 2010; Lutz et al., 2011; Hamid et al., 2014; Hamid et al., 2015; Taubert et al., 2018). Correspondingly, *E. tenella* and *E. necatrix* were demonstrated to trigger immunomodulatory molecules and NF-κB activation and to block apoptosis *via* Bcl-X_L_ during second merogony (del Cacho et al., 2004). Recently, we reported that *E. bovis* infection arrests endothelial cells in the G1-phase of the cell cycle and induces a premature senescence-like status in host cells (Velásquez et al, 2020b). Interestingly, senescent cells were reported to exhibit high metabolic activities and mitochondrial dysfunction (for review, see Sabbatinelli et al., 2019). The purpose of the present work is to analyze the energetic metabolism of *E. bovis*–infected primary bovine host endothelial cells and to explore the respective status of premature senescence observed in this host cell type.

## Materials and Methods

### Primary Bovine Umbilical Vein Endothelial Cell (BUVEC) Isolation and Maintenance

BUVEC were isolated from umbilical veins obtained from calves born by *sectio caesarea* at the Justus Liebig University Giessen, Germany. Therefore, umbilical cords were kept at 4°C in 0.9% HBSS-HEPES buffer (pH 7.4; Gibco, Grand Island, NY, USA) supplemented with 1% penicillin (500 U/ml; Sigma, St. Louis, MO, USA) and streptomycin (500 μg/ml; Sigma) for a maximum of 16 h before use. For the isolation of endothelial cells, 0.025% collagenase type II (Worthington Biochemical Corporation) suspended in Pucks solution (Gibco) was infused into the lumen of ligated umbilical veins and incubated for 20 min at 37°C in a 5% CO_2_ atmosphere. After gently massaging the umbilical veins, the cell suspension was collected and supplemented with 1 ml fetal calf serum (FCS, Gibco) to inactivate collagenase. After two washes (350*g*, 12 min, 20°C), cells were suspended in complete endothelial cell growth medium (ECGM, PromoCell, supplemented with 10% FCS), plated in 25 cm^2^ tissue plastic culture flasks (Greiner), and kept at 37°C in 5% CO_2_ atmosphere. BUVEC were cultured in modified ECGM medium (EGCM, diluted at 30% in M199 medium, supplemented with 5% FCS [Greiner} and 1% penicillin and streptomycin) applying medium changes every 2 to 3 days. BUVEC cell layers were used for infection after threee passages *in vitro*. All BUVEC isolations were conducted following the Institutional Ethics Commission of Justus Liebig University of Giessen, Germany, and by the current European Animal Welfare Legislation: ART13TFEU.

### *Eimeria bovis* Oocyst Production

The *E. bovis* strain H used in the present study was originally isolated in the field in northern Germany and has been maintained since then by passages in parasite-free Holstein Friesian male calves. For oocyst production, calves (*n* = 3) were orally infected at the age of 10 weeks with 3 × 10^4^ sporulated *E. bovis* oocysts. Experimental infections were conducted under the Institutional Ethics Commission of the Justus Liebig University of Giessen, Germany (allowance no.: JLU 589_AZ). Excreted oocysts were isolated from the feces at 18 days p. i. according to the method of Jackson (1964). Sporulation of oocysts was achieved by incubation in a 2% (w/v) potassium dichromate (Merck) solution at room temperature (RT). Sporulated oocysts were stored in this solution at 4°C until further use. Sporozoites were excysted from sporulated oocysts as previously described (Hermosilla et al., 2002). Free sporozoites were washed three times in sterile phosphate-buffered solution (PBS), suspended in complete Iscove’s modified Dulbecco medium (IMDM; Gibco), and counted in a Neubauer hemocytometer as described elsewhere (Hermosilla et al., 2008). Sporozoite viability was determined by trypan blue exclusion tests according to Lang et al. (2009).

### Host Cell Cultures and Parasite Infection

Depending on the experiment, BUVEC (three biological replicates) were either seeded in µ-dishes of 35-mm diameter (IBIDI^®^, Martinsried, Germany) for 3D-holotomographic microscopy (3D Cell Explorer-Fluo^®^, Nanolive), in Seahorse XFp^®^ Cell Culture Mini-plates (Agilent) for extracellular flux analysis or in 25 cm^2^ flasks (Greiner Bio-one) for NADPH measurements. In all cases, BUVEC were cultured at 37°C and 5% CO_2_ atmosphere until confluency. Then, cells were infected with 1.1 × 10^4^ (Seahorse^®^ 8-well plates), 3.5 × 10^4^ (µ-dishes of 35 mm) or 2.5 × 10^5^ (25 cm^2^ flasks) freshly excysted sporozoites. Cell culture medium was changed 1 day after infection and thereafter every third day. Infection rates were determined at 1 day p. i. microscopically. All plates (non-infected and *E. bovis*–infected) were seeded and infected at the same time to reduce the influence of experimental artifacts.

### Live-Cell 3D-Holotomographic Microscopy

Refractive index (RI)-based 3D-holotomographic images were obtained by a 3D Cell Explorer-Fluo^®^ (Nanolive) microscope equipped with a 60× magnification (λ = 520 nm, sample exposure 0.2 mW/mm^2^) and a depth of field of 30 µm. Therefore, non-infected and *E. bovis*–infected BUVEC samples (n = 3) were mounted on a top-stage incubator (IBIDI^®^, Martinsried, Germany) to control temperature, humidity, and CO_2_ levels. Images were processed using STEVE^®^ software (Nanolive) to obtain RI-based z-stacks (3D rendering). To further analyze the data, changes in parasitic structures over time were followed using a 3D-reconstruction of the Z-series displayed as maximum z-projections. Brightness and contrast were equally applied for compared image sets using Fiji software (Schindelin et al., 2012). Live cell imaging of mitochondria and mitochondrial membrane potentials (MMP) was performed using a fluorogenic mitochondrial staining for live cells, which is potential-independent (MitoView Green, Biotium). This probe accumulates in the mitochondria, giving a green fluorescence signal and labeling the whole mitochondrial network. The MMP was recorded in cells pre-loaded with Image-iT TMRM^®^ reagent which labels cells in red when mitochondria perform oxidative phosphorylation to produce ATP (ThermoFisher). The co-localization of total mitochondria-derived and MMP signals indicated those parts of mitochondria which are active in ATP production (light orange). The ratio between total mitochondrial network signals and the membrane potential was determined by calculating the total raw integrated density of FITC and TRITC channels per cell (Image J^®^; Fiji Software). The total area of cells was measured manually to avoid artifacts in the latest parasitic stages.

### Real-Time Analyses on Bioenergetics

For Seahorse XFp^®^-based experiments, BUVEC (*n* = 3) were grown in triplicates in eight-well Seahorse XFp^®^ Cell Culture Mini plates (Agilent) until confluence. Subsequently, BUVEC were infected and the cell culture medium (ECGM, PromoCell, supplemented with 10% FCS) was replaced by a fresh medium every two days. Here, two different measurements were performed: *i)* analysis of key mitochondrial function parameters using the Seahorse XFp^®^ Cell Mito Stress Test (Agilent) at 4 h, and 4, 8, 12, and 18 days p. i. and *ii)* analysis of glycolytic responses applying Seahorse XF^®^ Glycolysis Stress Test (Agilent) at the same days p. i. Both tests were performed in non-infected cultures to serve as controls and *E. bovis*–infected cultures in parallel following the manufacturer’s instructions. Briefly, for the Cell Mitostress test, BUVEC were incubated in assay-included medium [XF base medium (Agilent) supplemented with 25 mM glucose, 2 mM glutamine, and 1 mM sodium pyruvate; pH 7.4] for 60 min at 37°C under non-CO_2_-supplemented conditions. Oligomycin, rotenone/antimycin A, and FCCP (Carbonyl cyanide 4-(trifluoromethoxy)phenylhydrazone) were injected sequentially *via* instrument-own injection ports to block mitochondrial activity. Thus, data on basal mitochondrial respiration were obtained by the last rate measurement before first injection with oligomycin (inhibition of ATP synthase), minus the minimum rate measured after rotenone/antimycin A injection (complexes I, III, and ATP synthase inhibition). FCCP is an uncoupling agent that collapses the proton gradient and disrupts the mitochondrial membrane potential. It was used to obtain data on maximal mitochondrial respiration. Finally, data on non-mitochondrial oxygen consumption were generated by treatments with rotenone/antimycin A, a complex I/III that entirely blocks mitochondrial respiration. For the Glycolysis Stress test, the medium was also replaced by an assay-included medium (XF base medium DMEM supplemented with 2 mM glutamine) and BUVEC were incubated for 60 min at 37°C under non-CO_2_-supplemented conditions. Glucose, oligomycin, and 2-DG were sequentially injected to measure key parameters of the glycolytic function. Glucose was loaded in a saturating concentration of 10 mM to ensure full activation of the glycolysis pathway, a step necessary to obtain extracellular acidification rates (ECAR). Oligomycin inhibits mitochondrial ATP production and forces energy production toward glycolysis to obtain data on the maximum glycolytic capacity of the cells, and, finally, 2-DG (competitive inhibitor of hexokinase) treatments were applied to calculate the cellular glycolytic reserve. To control the quality of the cell monolayer and for cell number normalization, images were randomly taken before and after the measurements. The results obtained with Mitostress and Glycolysis stress kits for *E. bovis*–infected culture corresponds to an average value of the host cell as well as the parasite metabolism. To assess changes in the number of cells over the time of infection, the total cell number was determined previously in each assay. The difference in cell number was no more than 9.3% at 22 d p.i.

### Quantification of Metabolic Conversion Rates in Cell Culture Supernatants

Three biological replicates of BUVEC were grown in six-well plates (Greiner Bio-One) pre-coated with fibronectin (2.5 µg/μl, Sigma-Aldrich). At confluence, cells were infected with 1.9 × 10^5^ *E. bovis* sporozoites per well, and infection rates were controlled microscopically at 1 day p. i. For controls, non-infected BUVEC cells were cultivated in parallel. At 48 h p. i., the medium of infected as well as uninfected cells was entirely removed and replaced by exactly 2 ml of fresh medium. Subsequently, the cells were incubated at 37°C and 5% CO_2_ atmosphere for 48 h and, thereafter, cell supernatants from uninfected and infected cells were collected, centrifuged (400*g*, 10 min, 4°C), immediately frozen in liquid nitrogen and stored at −80°C for further analysis. In parallel, the corresponding numbers of cells/well for each supernatant sample were determined. This procedure was performed on days 4, 8, 12, 18, and 24 p. i. For metabolite concentration assessment, the frozen samples were heated (15 min, 80°C) and centrifuged (8,000*g*, 10 min). Concentrations of glucose, pyruvate, lactate, glutamine, glutamate, serine, and alanine were determined using a benchtop random access clinical chemistry analyzer as previously described in Mazurek et al. (2001). The conversion rates of individual metabolites were determined in nanomoles per h (h × 10^6^ cells) using medium samples without cells, which were processed as a medium reference in parallel to the cells. The supernatant from *E. bovis*–infected culture represents the combination of the metabolic signature from the host cell and the parasites.

### ROS Production and NADPH Measurements

Intracellular ROS production was measured in *E. bovis*–infected BUVEC at 4, 8, 12, 17, 21, and 24 days p. i. by 2,7-dichlorodihydrofluorescein diacetate (DCFH-DA) staining. The hydrolysis of the diacetate moieties in DCFH-DA produces 2,7-dichlorodihydrofluorescein (DCFH) that will be oxidized to DCF by oxidant species, such as those formed by H_2_O_2_ and peroxidases. Therefore, non-infected and *E. bovis*–infected BUVEC were incubated in 10 μM DCFH-DA for 30 min and thereafter washed with PBS (1×). A microplate reader Varioskan Flash^®^ (Thermo Scientific) at excitation/emission wavelengths of 485 nm/535 nm measured fluorescence intensities of the samples, respectively. To assess changes in the number of cells over the time of infection, the total cell number was previously counted in each assay. The difference in cell number was no more than 9.3% at 22 d p.i. Extracellular ROS production was estimated at 4, 8, 12, 17, 21, and 24 days p. i., by testing supernatants from *E. bovis*–infected BUVEC and non-infected controls (see *Quantification of Metabolic Conversion Rates in Cell Culture Supernatants*) using the Amplex Red^®^ reagent (ThermoFisher). The Amplex Red^®^ reagent is a highly sensitive and stable probe for H_2_O_2_ detection. It reacts at 1:1 stoichiometry with H_2_O_2_ to produce the fluorescent compound resofurin when it is in the presence of peroxidase, which is then detected by spectrofluorescence using 530/590 nm excitation/emission wavelengths, respectively. Supernatants from non-infected and *E. bovis*–infected cells were incubated in 41.5 µM Amplex Red^®^ and 7.5 U/ml HRP, and fluorescence was measured in a microplate reader (Varioskan Flash^®^, Thermo Scientific).

For NADPH measurements, BUVEC were seeded into 25 cm^2^ flasks to obtain a large number of cells (*n* = 3). The cell pellet from non-infected and *E. bovis*–infected culture were collected at 4, 8, 12, 17, 21, and 24 days p. i., centrifuged (10,000*g*, 10 min), and filtered through 10 kDa molecular weight cut off filters (BioVision) to concentrate the samples. NADPH concentration was measured by NADP/NADPH quantitation colorimetric kit (BioVision) following the manufacturer’s instructions.

Because of the fact that a combination between endothelial cells and parasites (sporozoites or merozoites depending on the parasitic stages) exists in the *E. bovis*–infected culture, the results obtained for intra- and extracellular ROS, as well as intracellular NADPH correspond to an average for the individual responses of the host cell and the parasite.

### Parasite Egress Induced by 2-DG and FDG Treatments

Three biological replicates of BUVEC were seeded in a 12-well plate (Greiner) and grown until confluence was achieved. BUVEC were infected with *E. bovis* sporozoites (3.5 × 10^5^ sporozoites per well) and cultured at 37°C and 5% CO_2_. Non-treated, *E. bovis*–infected cells were used as controls. Three different experimental approaches were tested to evaluate the effect of glycolytic pathway block on infected cells: *i*) at the trophozoite/early meront stage at day 8 p. i., cells were cultured in the presence of 2 mM 2-DG or FDG. Therefore, the medium containing 2-DG/FDG was replaced every 2 days. The meront area was evaluated over time to detect changes in the parasite development; *ii*) using the same cells in (*i*), the number of merozoite I production was analyzed by qPCR at 18 days p. i. Therefore, supernatants containing free-released merozoites I were harvested at 18 days p. i. and centrifuged at 1,500*g* for 10 min. Then, the pellet was suspended in 200 μl of PBS and total DNA was extracted using DNeasy Blood and Tissue Kit^®^ (QIAGEN) following the manufacturer’s instruction. Merozoite I production was measured by qPCR using specific primers for *E. bovis* microneme 4 (MIC4) gene (Hamid et al., 2014). In brief, the following primers and probes for EbMIC4 were used: forward primer 5′-CACAGAAAGCAAAA GACA-3′, reverse primer 5′-GACCATTCTCCAAATTCC-3′, and probe 5′-FAM-CGCAGTCAGTCTTCTCCTTCC-BHQ1-3′. A total volume of 25 μl was used for real-time PCR, containing 5 μl DNA, 20 μl PCR reaction mixture containing 10 μl PerfeCTa Fast Mix^®^ (Quanta, MD, USA), 400 μM of each primer, and 200 μM of the probe. The PCR conditions were initial denaturation at 95°C for 5 min followed by 45 cycles at 94°C for 15 s, 60°C for 45 s, and 72°C for 20 s. PCR amplification was performed on a Rotor-Gene Q^®^ cycler (Qiagen) with the acquisition in the green channel. For the quantification of merozoite I counts in the samples, DNA from a dilution series of known merozoite I numbers (16; 160; 1,600; 16,000; 160,000; 1,600,000) was included. The standard curve was generated by plotting EbMIC4-Ct values against the logarithm of the number of merozoites (Hamid et al., 2014); *iii*) and the short-term effect that 2-DG or FDG treatments had on infected cells was subsequently tested. At 4, 8, 12, and 18 days p. i., infected cells were treated with 2 mM 2-DG or FDG for 2 h and the number of egressed intracellular sporozoites were counted in a Neubauer chamber. The concentrations of 2DG and FDG, as well as the incubation times were previously probed for coccidian parasites by Taubert et al., 2016.

### Statistical Analysis

All data were expressed as mean ± SD from three independent experiments. Analysis of the metabolic parameters and ROS production during time (kinetics) was achieved by performing a two-way ANOVA analysis with repeated measurements defining the time as the matching variable. Significance between the infected and non-infected group was estimated by a Bonferroni multiple comparison test. The merozoites production graph was analyzed with a multiple comparisons one-way ANOVA. Significance was defined as p ≤ 0.05. All graphs and statistical analyses were performed using GraphPad Prism^®^9 software.

## Results

### *Eimeria bovis* Macromeront Formation Triggers Glycolytic Responses and Alters Metabolic Signatures of Host Endothelial Cells

*E. bovis* is an intracellular parasite that exclusively develops in bovine endothelial cells *in vivo*. Previous results from our group showed that *E. bovis* sporozoites successfully grow in BUVEC and producing mature macromeronts from days 17 p. i. onward. Thus, *E. bovis* considerably differs from other apicomplexan members by its long-lasting intracellular merogony I where infected host endothelial cells may reach 300 μm or more in size.

**Figure 1:**
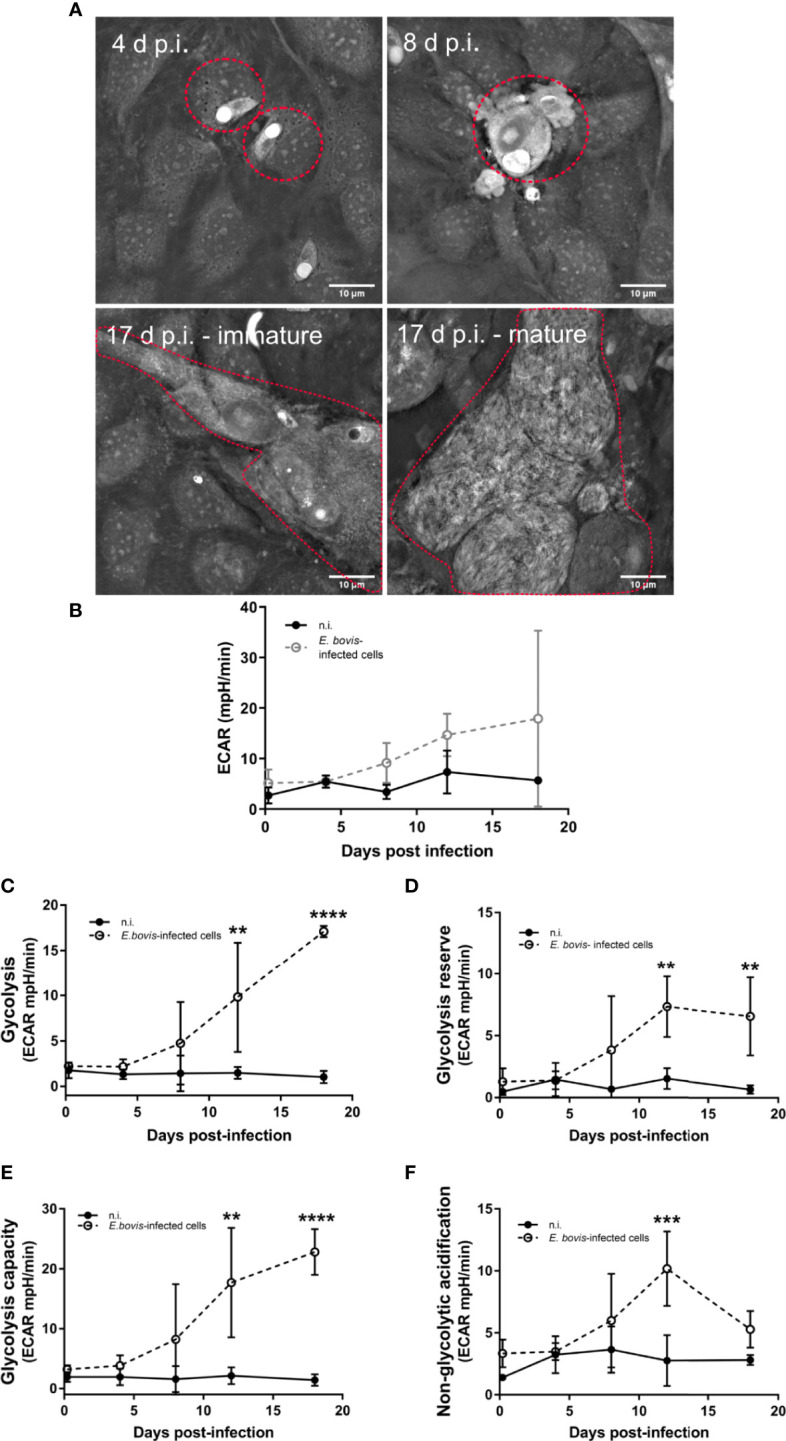
Analysis of the glycolytic metabolism during the *E. bovis in vitro* development in endothelial cells. **(A)** Confluent primary endothelial cells *(n = 3)* were infected with freshly isolated *E. bovis* sporozoites. From 4 days p. i. onward, the same cell layer was monitored microscopically to evaluate parasite development. 3D-holotomographic microscopy used refractive indexes (RI) of cell components to reconstruct and visualize the total cell and meront structures over time. Premature and mature meronts I structure were observed from 17 days p. i. **(B–E)** BUVEC were infected with *E. bovis* sporozoites and tested for glycolytic responses by a glycolysis stress test (Agilent). After sequential supplementation of 10 mM glucose, 1 μM oligomycin, and 50 mM 2-DG, several glycolytic parameters were measured, in *E. bovis*–infected (◌) and non-infected cells (•) at the indicated days p. i. ECAR **(B)**, glycolysis **(C)**, glycolysis reserve **(D)**, glycolysis capacity **(E)**, and non-glycolytic acidification **(F)** were determined at 4 h p. i. and 4, 8, 12, and 18 d p. i. Statistical analysis was performed by using two-way ANOVA with repeated measurements defining the time as the matching variable (significance defined as p ≤ 0.05). Values are expressed as means ± SD of three biological replicates. **p ≤ 0.01, ***p ≤ 0.001, ****p ≤ 0.0001.

The main aim of the current study was to assess metabolic responses and mitochondrial function in *E. bovis*–infected host endothelial culture. Using a 3D-holotomographic live cell-based imaging system, we detected different developmental stages during macromeront formation, i. e. intracellular sporozoites, trophozoites, immature/mature macromeronts, and merozoites I (**Figure 1A**), thus confirming successful parasite development in BUVEC.

An extracellular flux analyzer Seahorse XFp assessed the glycolytic profile of non-infected and *E. bovis*–infected BUVEC. The glycolysis stress kit test uses glucose, oligomycin, and 2-DG (deoxyglucose) supplementation to calculate several glycolytic parameters, such as total glycolysis, glycolytic capacity, and glycolytic reserve, based on ECAR (**Figure 1B**). This experimental approach allowed us to perform sequential measurements on metabolic changes during *E. bovis* first merogony in living BUVEC culture. Glucose supplementation (10 mM) induced a time-dependent increase of ECAR as a result of increased glycolysis being significantly higher in *E. bovis*–infected culture from 12 days p. i. onward compared with equally processed control cells (12 days p. i.: *p* = 0.0018, 18 days p. i.: *p* < 0.0001) (**Figure 1C**). Likewise, glycolytic reserve and capacity were found significantly increased in *E. bovis*–infected culture from 12 and 19 days p. i., onward (**Figures 1D, E**) (glycolytic reserve: 12 and 19 days p. i.: *p* = 0.0092 and *p* = 0.0081; glycolytic capacity: 12 and 19 days p. i.: *p* = 0.00032 and *p* = 0.0001). Both parameters are in direct correlation with the later stages of intracellular parasite development. As an interesting finding, treatments of *E. bovis*–infected culture with the glucose analogue 2-DG—which inhibits glycolysis—revealed that *E. bovis* infection induced non-glycolytic pathways up-regulation in the infected monolayer, leading to enhanced proton efflux in infected culture (**Figure 1F**, *p* = 0.0007).

**Figure 2:**
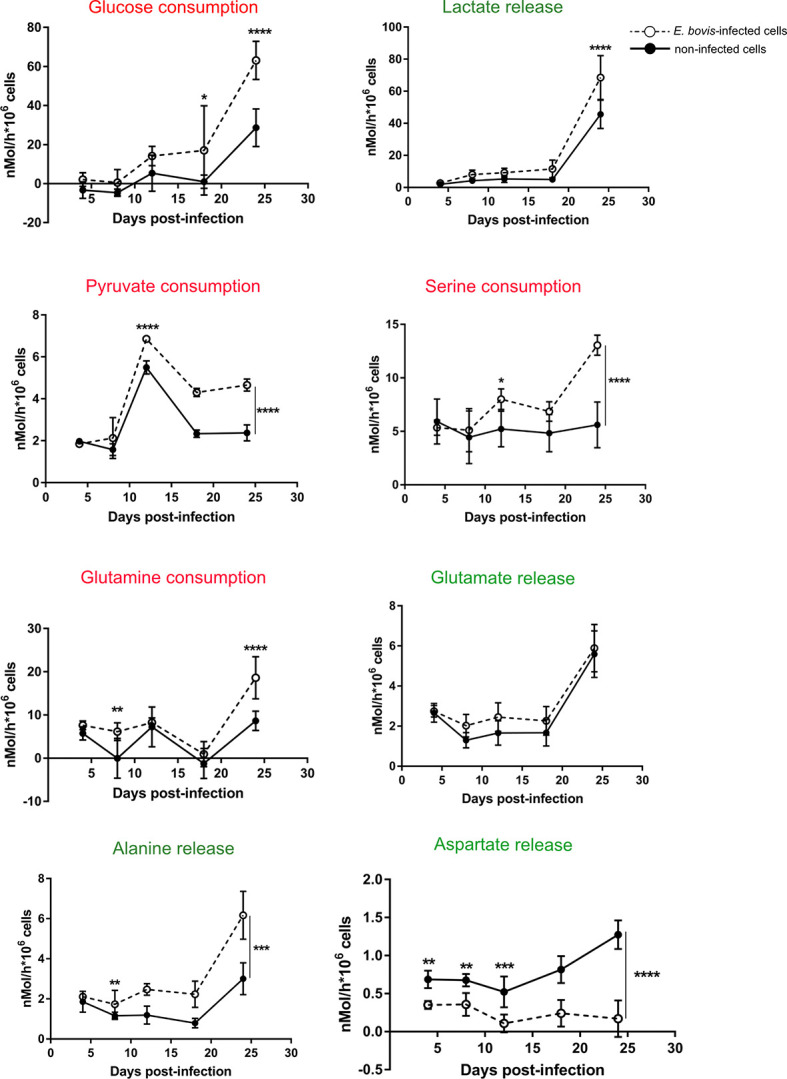
*E. bovis* infection modulates metabolic responses in host endothelial cells. The metabolic signatures of non-infected and *E. bovis*–infected BUVEC were measured at 4 h p. i., 4, 8, 12, 18, and 24 days p. i. The concentration of several key molecules related to glucose metabolism as well as glutaminolysis, another important source of energy and cell building blocks were determined in supernatants of non-infected and *E. bovis*–infected cells at each time point. The metabolite conversion rates were normalized by the total number of cells present in the cell layer at each condition. The results are represented as nMol/h per 1 x10^6^ cells of each metabolite, in *E. bovis*–infected (◌) and non-infected cells (•) at the indicated days p. i. Statistical analysis applied was a two-way ANOVA with repeated measurements defining the time as the matching variable (significance defined as α ≤ 0.05). Values are expressed as means ± SD of three biological replicates. *p = 0.05, **p ≤ 0.01, ***p ≤ 0.001, ****p ≤ 0.0001.

To investigate how *E. bovis* is modulating the glycolytic activity of infected host cell culture, we quantified the metabolic conversion rates of key products from carbohydrate and amino acid metabolism in cell culture supernatants of non-infected and *E. bovis*–infected cultures. Cell culture supernatants of *E. bovis*–infected host cells were collected from 4 to 18 days p. i. and conversion rates of glucose, pyruvate, lactate, serine, alanine, glutamine, glutamate, and aspartate were quantified relative to plain medium without cells cultivated in parallel to the dishes with cells. Glucose, serine, pyruvate, and glutamine consumption increased over time during infection (**Figure 2**). Likewise, lactate and alanine production was upregulated at the endpoint of infection while aspartate production decreased. The rate of glutamate release remained unchanged in E. *bovis*–infected culture in comparison to uninfected controls (**Figure 2**). A summary of all parameters measured in *E. bovis*–infected host cell cultures is displayed in **Table 1**.

**Table 1:** Summary of the metabolic measurements on *E. bovis*–infected BUVEC.

	*E. bovis*–infected BUVECs					
days p. i.	4 h p.i.	4	8	12	17–19	24
Stages of macromeront formation	Sporozoite		Trophozoite	Immature macromeront		Mature macromeront
glucose consumption	–	n.s.	n.s.	n.s.	↑*	↑****
lactate production	–	n.s.	n.s.	n.s.	n.s.	↑****
serine consumption	–	n.s.	n.s.	↑*	↑n.s	↑****
pyruvate consumption	–	n.s.	n.s.	↑****	↑****	↑****
glutamine consumption	–	n.s.	↑**	n.s	n.s.	↑****
glutamate production	–	n.s.	n.s.	n.s.	n.s.	n.s.
alanine production	–	n.s.	↑**	↑***	↑***	↑***
aspartate production	–	↓**	↓**	↓***	↓****	↓****
**Glycolysis stress assay**						
Glycolysis	n.s.	n.s.	↑n.s.	↑**	↑****	–
glycolysis reserve	n.s.	n.s.	↑n.s.	↑**	↑**	–
glycolysis capacity	n.s.	n.s.	↑n.s.	↑**	↑****	–
ECAR	n.s.	n.s.	↑n.s.	↑***	↑n.s.	–
non-glycolytic acidification	n.s.	n.s.	↑n.s.	n.s.	↑n.s.	–
**Mitostress assay**						
basal respiration	n.s.	n.s.	n.s.	↑n.s.	n.s.	–
maximal respiration	n.s.	n.s.	n.s.	↑n.s.	n.s.	–
ATP production	n.s.	n.s.	n.s.	n.s.	n.s.	–
Non-mitochondrial oxygen consumption	n.s.	n.s.	n.s.	↑**	↑**	–
extracellular ROS	–	↑*	n.s.	n.s.	n.s.	↓n.s.
intracellular ROS	–	n.s.	↑****	↑****	↑n.s.	↑****
NADPH	–	n.s.	n.s.	n.s.	n.s.	↑*

^1^The vertical arrow indicate an increased activity of the measure pathway.

### Inhibition of Glucose Catabolism Delay Parasite Development and Boosts Sporozoite Egress From Host Cells

**Figure 3:**
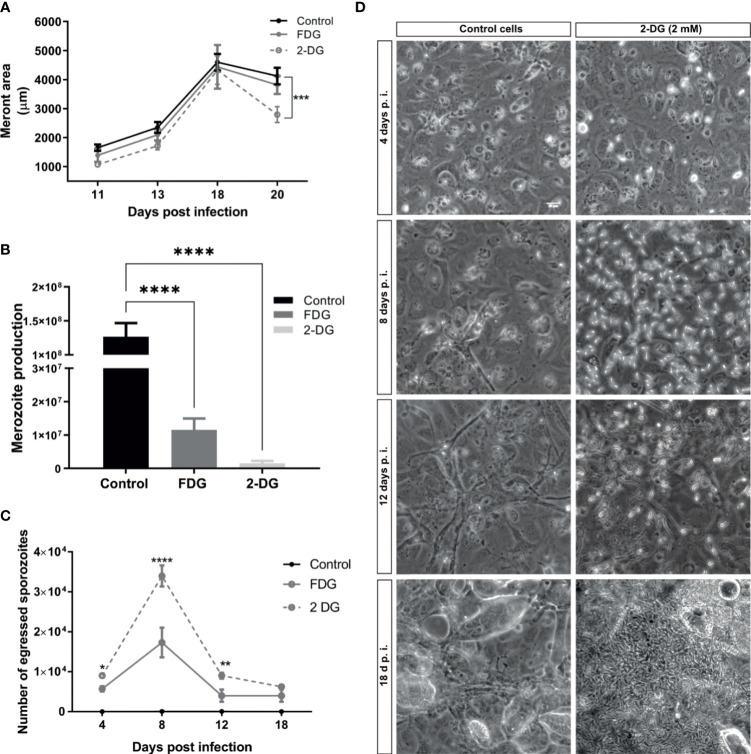
2-DG and FDG treatments diminish merozoite I production and induce *E. bovis* sporozoites egress. **(A)** *E. bovis* –infected cells (n = 3) were treated with 2 mM 2-DG or FDG from day 8 p. i. onward. As a read-out, the meront area was evaluated at 11, 13, 18, and 20 days p. i. to detect changes in meront growth. **(B)** At days 18 p. i., supernatants of *E. bovis*–infected cells treated with 2 mM 2-DG or FDG were analyzed for the presence of merozoites I *via* EbMIC4-specific qPCR to estimate merozoite I production and release. *E. bovis*–infected cells lacking any treatment were processed in parallel and served as controls. **(C)** Analysis of short-term effects of 2-DG or FDG treatments on *E. bovis*–infected cells. Infected cells were treated at 4, 8, 12 at 18 days p. i. with 2 mM 2-DG or FDG for 2 h. The number of sporozoites released into the cell culture supernatants within 10 min was counted; representative images are illustrated in **(D)**. Important to notice that the image on **(D)** for cells treated with 2-DG at 18 d p. i. shows mainly merozoites but not sporozoites releaset to the extracellular space. Statistical analysis applied was a two-way ANOVA with repeated measurements defining the time as the matching variable for A and **(C)** For B, the statistical test was a multiple comparisons one-way ANOVA (significance defined as α ≤ 0.05). Values are expressed as means ± SD of three biological replicates. **p ≤ 0.01, ***p ≤ 0.001, ****p ≤ 0.0001.

It is well known that obligate intracellular parasites scavenge glucose from their host cells. The results of the glycolytic stress test, as well as the direct measurement of nutrients and metabolic products in cell culture medium, suggest a pivotal role of glycolysis for successful *E. bovis* macromeront formation. To better understand the role of glucose in *E. bovis* macromeront formation, we additionally studied the impact of long-term glycolysis inhibition on parasite development (4–18 days p. i.). Therefore, infected BUVEC were treated with the glucose analogues 2-deoxy-d-glucose (2-DG) and fluoro 2-deoxy-d-glucose (FDG) from 8 days p. i. onward. The meront size was used as a measure of the parasite development, as well as the number of merozoites released after 18 days of infection. These two parameters are related to normal parasite development. Non-treated *E. bovis*–infected BUVEC served as a control condition. Current data show that the mean meront size was only significantly smaller in the case of 2-DG treatment at the late phase of merogony I (**Figure 3A**), but the total number of released merozoites I showed a significant reduction of 99% for 2-DG and 91% for FDG treatments at 20 days p. i. (*p* < 0.0001, **Figure 3B**). Overall, these data underline that the glycolysis blockage affects the meront size but also the merozoite I production, suggesting that glucose is important for successful *E. bovis* intracellular development. However, the results cannot assess whether the glycolysis inhibition of the parasites, host cell, or both explain the delayed parasite development. As a highly interesting finding of this experimental approach, we additionally observed that short-term treatments with both glycolysis inhibitors induced an egress of *E. bovis* sporozoites from host cells on days 4 to 12 p. i. (**Figures 3C, D**). This suggests that *E. bovis* sporozoites require an active glycolysis pathway within the host cell cytosol. In contrast, when the host cellular glycolytic activities diminish (these conditions being unfavorable for sporozoite development), the parasite triggers mechanisms to egress from the host cells. Considering the active nature of cellular egress, it seems unlikely that this behavior is a result of parasite-own glycolysis inhibition. However, it is a common observation for *E. bovis in vitro* cultures, that several sporozoites remain intracellular without further development but occasionally egress and re-infect other cells in the same cell layer later on. So far, the reasons for this spontaneous egress are unknown, but based on the current data, it is maybe linked to unsuitable metabolic conditions in individual host cells.

### *Eimeria bovis* Infection Triggers Mitochondrial Responses in Infected Host Cells

**Figure 4:**
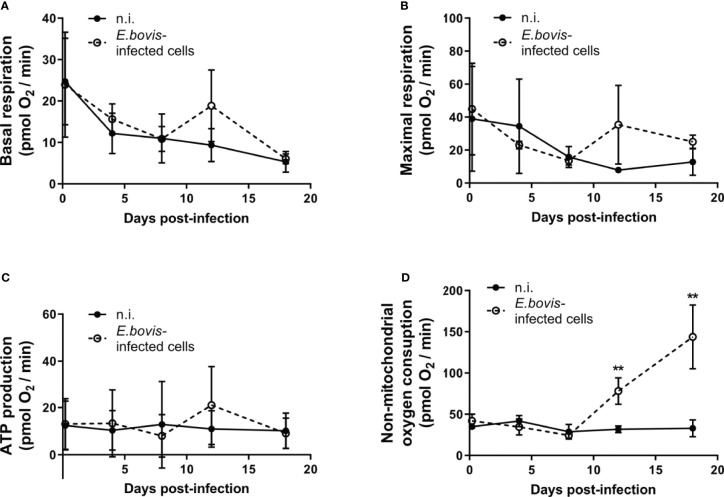
*E. bovis*-driven modulation of the host cellular carbohydrate catabolism. BUVEC (n = 3) were infected with *E. bovis* sporozoites and tested for mitochondrial responses by a mitochondrial stress test (Agilent). After the sequential supplementation of 1 μM oligomycin, 0.5 μM FCCP, and 1 μM rotenone/antimycin (*A*) several mitochondrial parameters were measured in *E. bovis*–infected (◌) and non-infected cells (•) at 4 h p. i., 4, 8, 12, 18 days p. i. Based on OCR values (pmol/min), single parameters, such as basal respiration **(A),** maximal respiration **(B)**, ATP production **(C)**, and non-mitochondrial oxygen consumption **(D)** were determined. [basal respiration = (last rate measurement before the first injection) − (non-mitochondrial respiration rate)]. Statistical analysis applied was a two-way ANOVA with repeated measurements defining the time as the matching variable (significance defined as *p ≤* 0.05). Values are expressed as means ± SD of three biological replicates. **p ≤ 0.01.

Glycolytic stress tests as well as the metabolic flux measurements additionally pointed to a non-glycolytic impact of *E. bovis* infection on host cells. Mitochondrial stress tests revealed no changes in basal and maximal respiration, as well as ATP production, between non-infected and *E. bovis*–infected cells (**Figures 4A–C**). In contrast, non-mitochondrial oxygen consumption—representing the oxygen consumption by cyclooxygenases, lipoxygenases, and NADPH oxidases—was increased after 12 days p. i. in *E. bovis*–infected cells whilst remaining constant over time in non-infected cells (**Figure 4D**).

### *Eimeria bovis* Infection Alters Host Cellular Mitochondrial Dynamics

**Figure 5:**
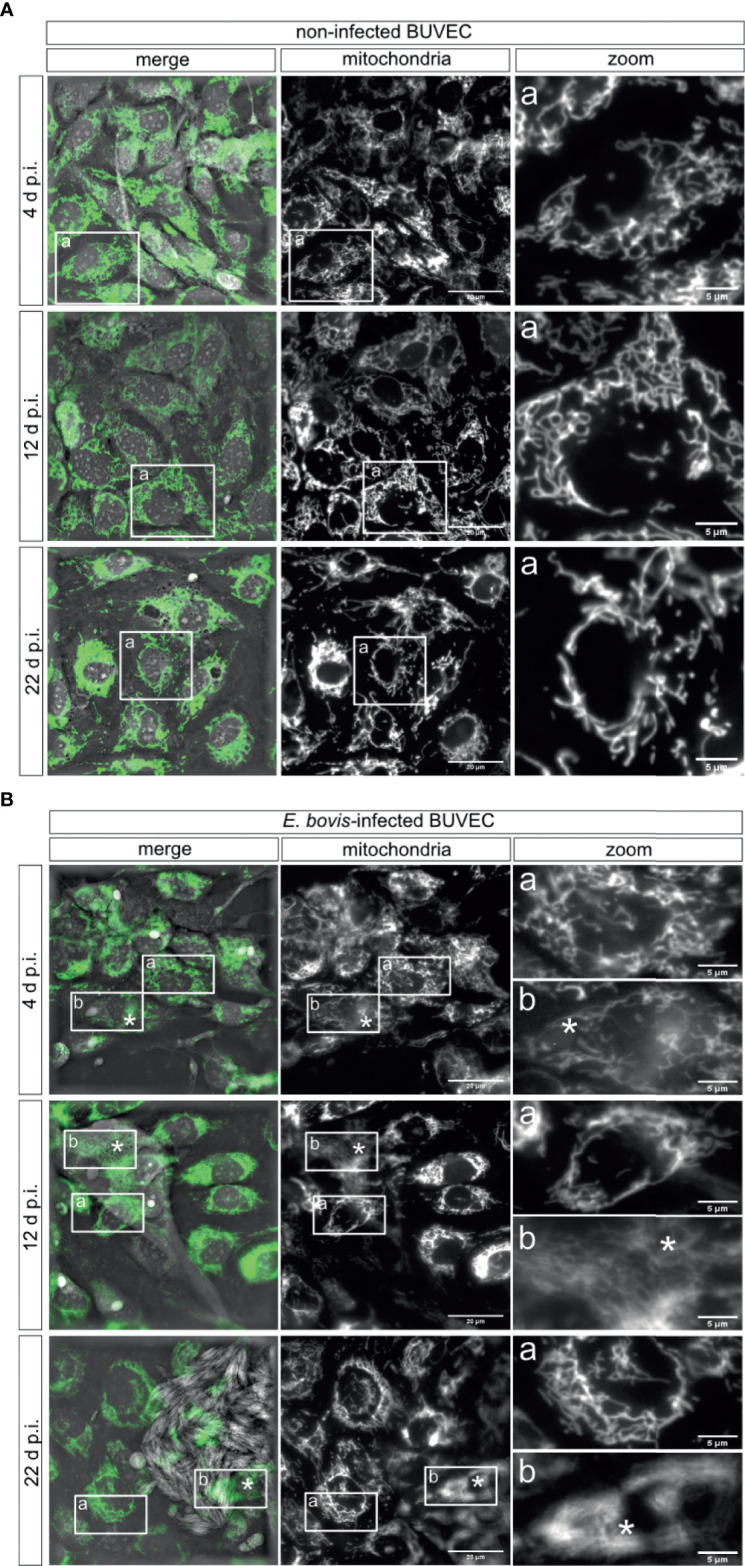
Mitochondrial morphology of *E. bovis*–infected host cells. Non-infected controls **(A)** and *E. bovis* –infected BUVEC (n = 3) **(B)** were stained with a mitochondrial probe independent on the mitochondrial potential (Mitoview, green) to visualize mitochondrial morphology. The mitochondrial structure (fluorescence) was registered at 4, 12, and 22 days p. i. The images are representative of three biological replicates and are shown in grayscale for visualization purposes. **(A-a)** Inset square in mitochondria and merge images are shown in the zoom panel to observe the mitochondria network in one cell. **(B-a, b)** Inset rectangles in mitochondria and merge images are shown in the zoom column to identify the mitochondria shape in non-infected cells **(a)** and *E. bovis*–infected cells **(b)** in only one cell. Asterisks identify the parasite localization in the image. *p = 0.05.

To investigate mitochondrial dynamics in *E. bovis*–infected BUVEC culture, we performed *in vivo* live cell imaging using 3D-holotomography in combination with fluorescence-based mitochondrial markers. *E. bovis*–infected and control BUVEC cultures were pre-loaded with mitochondrial probes detecting total mitochondria (green) and mitochondrial membrane potential (MMP, red). As expected, the classical mitochondrial morphology represented by a large interconnected network mainly radiating from the nuclear region with a mean transversal size of 0.379 ± 0.034 μm was observed in non-infected controls (**Figure 5A**). Overall, the mean mitochondrial thickness did not change in *E. bovis*–infected culture (0.395 ± 0.109 μm) even though, by tendency, a slightly shorter mitochondrial length was found in the first days p. i. However, when macromeronts matured, the shape of the mitochondrial network drastically changed. Thus, mitochondrial structures seemed less defined and showed a blurred appearance and a more filamentous organization (**Figure 5B-b**, 12 and 22 days p. i. denoted by asterisks). Of note, non-infected cells in the same cell layer directly neighboring *E. bovis*–infected cells (**Figure 5A-a**) presented a normal mitochondrial shape and distribution (**Figure 5B-a**).

**Figure 6:**
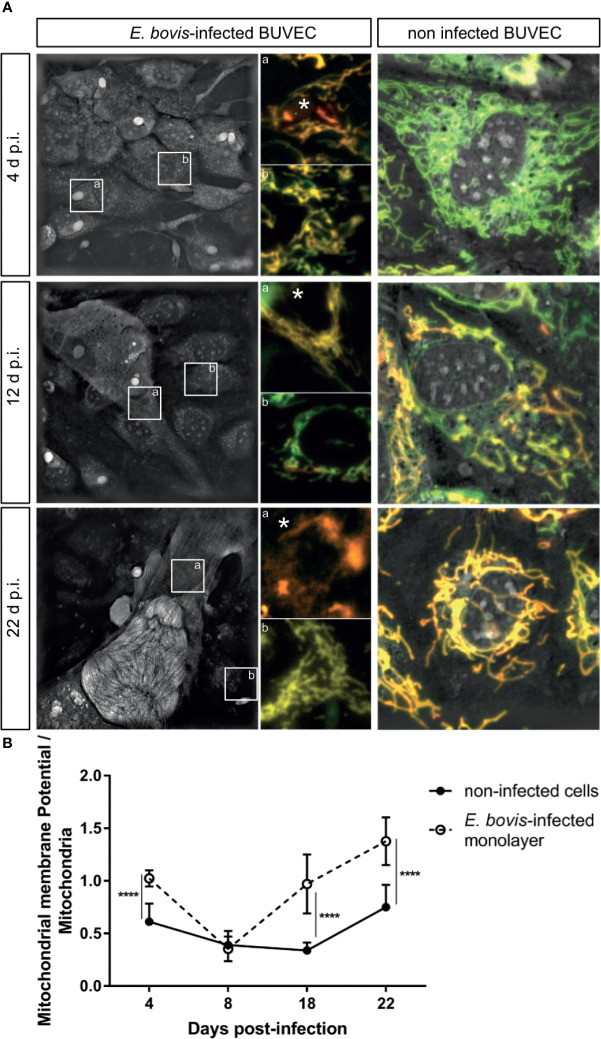
*E. bovis*-triggered host cellular mitochondrial membrane potential alteration. Mitochondrial membrane potential was evaluated by live-cell imaging using the Image-iT TMRM reagent in non-infected and *E. bovis* –infected cells. **(A)** 3D-holotomographic images based on refractive index (RI) were acquired to illustrate the entire mitochondrial network at 4, 12, and 22 days p. i. The insets in the RI image illustrate one infected cell **(a)** and a non-infected cell **(b)** within the same monolayer. **(a, b)** shows the co-localization of total mitochondria (green) with membrane potential (red). The non-infected BUVEC panel shows the co-localization of the 3D-holotomography and the two fluorescence channels: mitochondria (green) as well as the membrane potential (red) for the non-infected samples. **(B)** The ratios of total mitochondrial: mitochondrial membrane potential-related signals were estimated by fluorescence intensities in both non-infected and *E. bovis*–infected cells. Asterisks identify the parasite localization in the image. Statistical analysis applied was a two-way ANOVA with repeated measurements defining the time as the matching variable (significance defined as α ≤ 0.05). Values are expressed as means ± SD of three biological replicates. *p = 0.05, ****p ≤ 0.0001.

**Figure 7:**
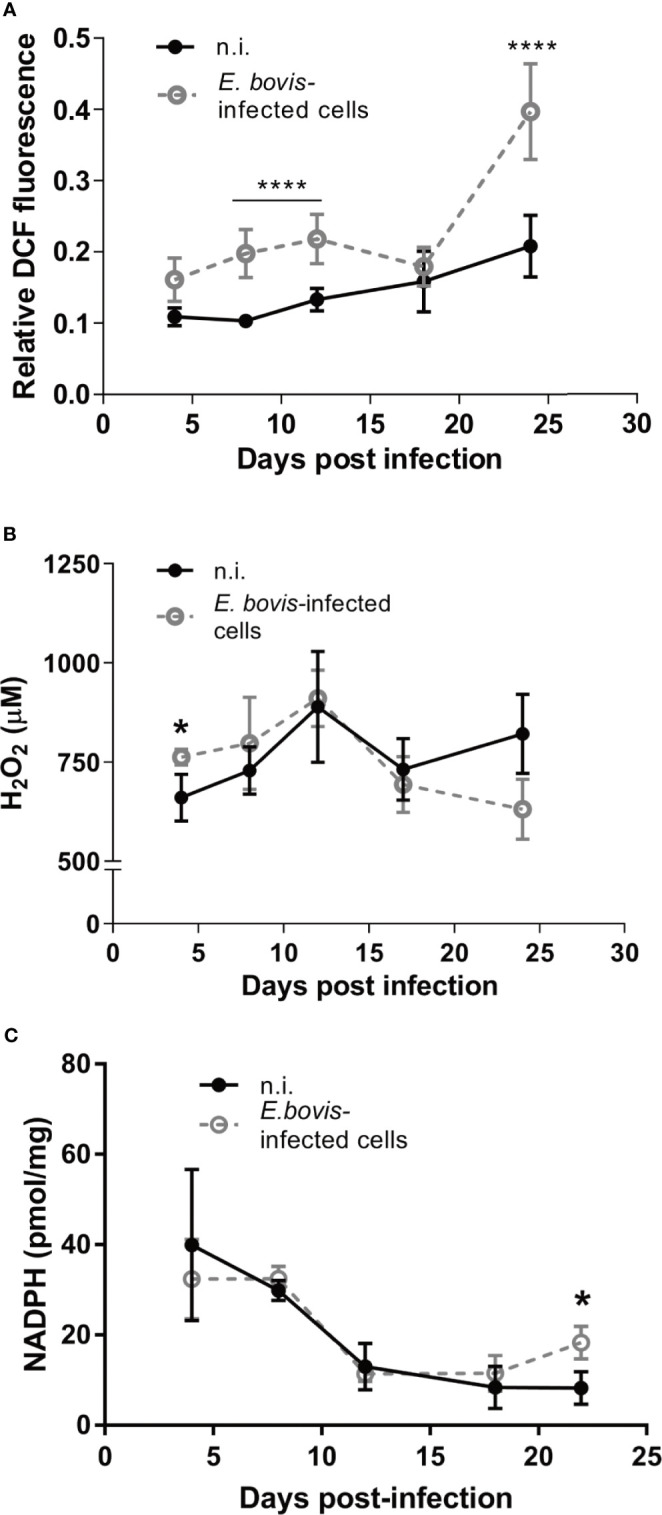
*E. bovis* infection boosts intracellular ROS production in the host cell. To evaluate the redox status in *E. bovis*–infected cells over time, intra- and extracellular ROS production and intracellular NADPH concentration were evaluated. **(A)** Intracellular ROS production was evaluated *E. bovis* –infected (◌) and non-infected cells (•) loaded with DCFH-DA. Fluorescence intensity was measured and the results were plotted as relative fluorescence units. **(B)** Supernatants from non-infected and *E. bovis* –infected BUVEC were collected between 5 and 24 days p. i. and H_2_O_2_ concentration was determined by Amplex Red-based tests. **(C)** The same monolayers used for the supernatant measurements, were collected and used to quantify intracellular NADPH concentrations. Statistical analysis applied was a two-way ANOVA with repeated measurements defining the time as the matching variable (significance defined as α ≤ 0.05). Values are expressed as means ± SD of three biological replicates. *p = 0.05, ****p ≤ 0.0001.

The simultaneous detection of mitochondria (green, non-potential dependent probe) and their membrane potential (MMP, red) was indicated by the colocalization of both signals (= light orange) in non-infected controls and *E. bovis*–infected cultures at 4 days p. i. (**Figure 6A**, 4 d p. i.-a). At 12 days p. i., non-infected bystander cells (located nearby *E. bovis*–infected cells) gave reduced MMP-derived red signals compared with 4 days p. i. (**Figure 6B**—12 days p. i.-b) whilst *E. bovis*–infected cells showed co-localization of both signals in mitochondria increasingly surrounding the meronts (**Figure 6A**, 12 days p. i.). At 22 days p. i., MMP signals were found enhanced suggesting that mitochondria of infected cells experienced highly active membrane potentials (**Figure 6A**—22 days p. i.). Overall, quantification of both signals in the same cell for ratio calculation revealed a significant rise in membrane potentials in *E. bovis*–infected BUVEC (**Figure 6B**). Mitochondrial damage or dysfunction may also lead to enhanced ROS production. To estimate whether *E. bovis*-driven mitochondrial alterations would also be reflected by this parameter, intracellular and extracellular ROS concentrations were estimated during the first merogony in *E. bovis*–infected cultures. Indeed, an increase in intracellular ROS production was detected at both early (12 days p. i.: *p* < 0.0001) and late merogony (**Figure 7A**—20–24 days p. i. with *p* < 0.0001) whilst extracellular ROS concentration was not influenced (except for 4 days p. i.) by parasite infection (**Figure 7B**). NADPH production, which is indicative of the redox state of a cell, was found altered exclusively at the mature macromeront stage (**Figure 7C**), thereby confirming a change in the metabolic status of the infected host cell culture at late macromeront development.

## Discussion

**Figure 8:**
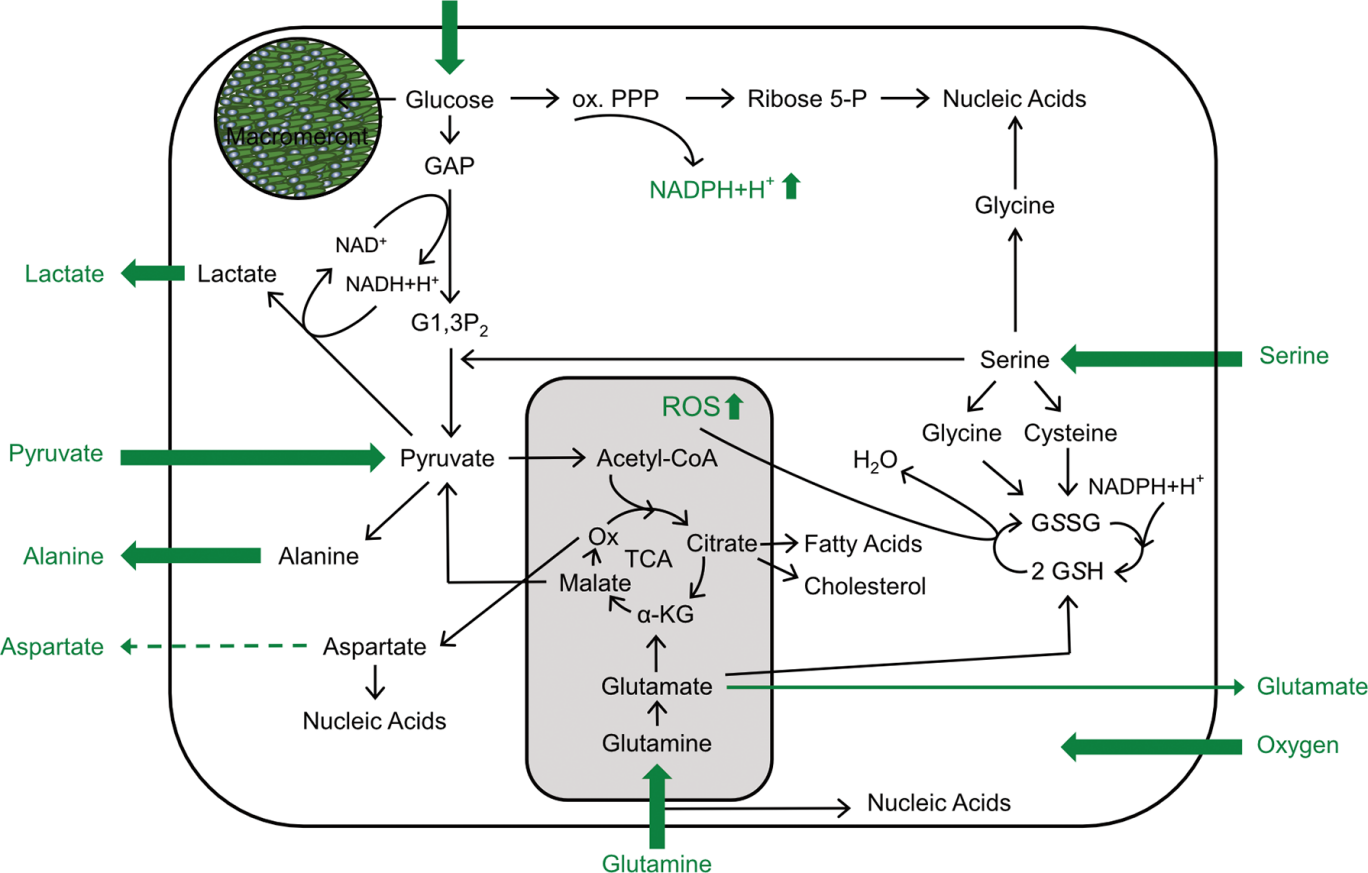
Scheme of *E. bovis*-driven metabolic modulations of the host cell. Impact of *E. bovis* on BUVEC metabolism. All metabolic parameters actually measured are shown in green. Thick arrows indicate an increase of the respective metabolic parameter; dashed arrows a decrease, and thin arrows unchanged metabolic parameters.

One of the most intriguing features of *E. bovis* development is the formation of intracellular macromeronts with sizes up to 300 μm within endothelial host cells (Hermosilla et al., 2008). Remarkably, the host cells survive and support this development by an intense cytoskeletal re-organization (Hermosilla et al., 2008). Full macromeront formation takes up to 25 days and thereby considerably exceeds the merogonies of other closely related apicomplexans, such as *T. gondii*, *Neospora caninum*, or *B. besnoiti* (Conejeros et al., 2019; Velásquez et al., 2019; Velásquez et al., 2020a; Velásquez et al., 2020b; Larrazabal et al., 2021). Besides cytoskeleton, *E. bovis* modulates several pathways and cellular functions, such as apoptosis, cholesterol biosynthesis, immune responses, or cell cycle progression to complete its intracellular development (Lang et al., 2009; Taubert et al., 2010; Hamid et al., 2015; Velásquez et al., 2020b). Metabolic requirements for intermediates products, such as amino acids, lipids or ATP molecules, can be achieved in a fast and efficient fashion when glucose is used as substrate. In line with this assumption, the current data revealed that *E. bovis* development boosted glycolytic responses in infected host cell culture. Remarkably, these metabolic changes only applied to late phase merogony, thereby indicating a stage-specific metabolic impact on infected host cells culture (for a summary, refer to **Figure 8** and **Table 1**).

*E. bovis* macromeronts exclusively develop in bovine endothelial cells, which typically use the anaerobic conversion of glucose to lactate for energy generation *via* ATP production (Sabbatinelli et al., 2019). Our results showed an increased glycolytic conversion rate in the infected cell culture that was several times higher compared with non-infected cells culture. The study of the current metabolic profiles of infected and control cells suggests *E. bovis*–infected BUVEC cultures degrade glucose to lactate, even in the presence of saturating oxygen concentrations. Increased conversion of pyruvate to lactate in *E. bovis*–infected cultures suggests a mechanism to ensure a high glucose conversion rate for successful parasite development (MacRae et al., 2012; Taubert et al., 2016). Lactate- and pyruvate-related kinetics showed pyruvate consumption to be increased before lactate production. This might indicate that pyruvate fuelled both the TCA cycle and lactate production. Supporting this hypothesis, pyruvate consumption increases at mature macromeront stages. The importance of glycolysis for the host cell intracellular *E. bovis* development was additionally tested by treatments with two glycolytic inhibitors, 2-DG and FDG, which significantly affect the merozoite I production as well as the meront size. As a highly interesting finding, we here additionally noticed that *E. bovis* sporozoite or the host cell may sense the glycolysis inhibition or the adverse intracellular conditions, increasing the number of sporozoites released from the host cells, shortly after 2-DG/FDG treatments. The fact that a certain proportion of sporozoites remain within host cells without any further development is a common finding in *E. bovis*–infected endothelial cell cultures. These sporozoites remain viable and may egress from their distinct host cell even three weeks after infection but usually re-invading other host cells within the cell monolayer. However, it was unknown that a glycolytic block in the infected host cell culture may trigger intracellular sporozoite egress. Interestingly, the transfer of egressed sporozoites from infected monolayers treated with glycolysis inhibitors to non-treated cell layers resulted in the formation of normal macromeronts. Thus, it seems that inhibitor treatments neither harm the parasite invasive nor improve developmental capacities. The hexokinase enzyme, the first enzyme in the glycolysis pathway, which is highly divergent from those of humans, animals, and parasites, which suggests that the parasite enzyme is not affected by 2DG or FDG treatment and, therefore, the parasite’s glycolytic pathway is not compromised.

We recently reported that *E. bovis* first merogony drives endothelial host cells into premature senescence (Velásquez et al., 2020b). Interestingly, a boosted glycolytic pathway is a typical feature of cellular senescence (Zwerschke et al., 2003; Unterluggauer et al., 2008). Former studies on cellular senescence showed a strong reduction in ATP production based on dysregulation of some glycolytic enzymes (Wang et al., 2003; Zwerschke et al., 2003; Unterluggauer et al., 2008), but no changes were observed in the ATP production in neither non-infected nor *E. bovis*–infected BUVEC culture. This may be explained by the high overlap of both decreased host cellular ATP production and/or parasite-related ATP consumption/production. Nevertheless, live-cell studies on mitochondrial morphology in *E. bovis*–infected cells revealed two phenotypes of mitochondria. Non-infected cells, even in the *E. bovis*–infected cell layers, showed a normal mitochondrial network surrounding the host cell nucleus. In contrast, mitochondria of *E. bovis*–infected cells appeared morphologically altered and exhibit a deteriorated phenotype that is characterized by a more diffuse, elongated, and filamentous morphology. Mitochondrial alteration correlated with ongoing macromeront maturation was exclusively observed in infected cells but never in bystander cells. Given that the mitochondrial morphology corresponds to an equilibrium between fusion and fission processes and responds to intracellular requirements (Bernhardt et al., 2015), we suggested that the parasite metabolism and/or localization can affect the dynamics of the host mitochondrial fusion-fission process. However, further research and experimentation should be done to assess the role of host cell mitochondria in the *E. bovis*–infected cells.

Some reports on the relationship between mitochondrial shape and its function stated that progressive mitochondrial elongation was linked to drug-induced senescence and accompanied by OXPHOS complex II defects (Kwon et al., 2019). Moreover, OXPHOS deterioration is involved in the early stages of cellular senescence (Yoon et al., 2003; Yoon et al., 2005; Kwon et al., 2019). Current observations may therefore agree with a previous report on *E. bovis*-driven pre-mature senescence in host cells (Velásquez et al., 2020b). Contradictory to our observations in which the mitochondrial membrane potential increased in *E. bovis*–infected cells, senescent cells have been describing to reduce the MMP as a consequence of the mitochondrial malfunction (Korolchuk et al., 2017; Chapman et al., 2019). However, more detailed analyses are needed to understand how the level of MMP increase in infected cells could be related to the senescence process induced by parasite infection. Interestingly, mitochondrial dysfunction may also trigger cellular senescence by increased production of ROS (Passos et al., 2007; Moiseeva et al., 2009; Velarde et al., 2012; Wiley et al., 2016). Enhanced levels of ROS are directly related to damage to DNA, proteins, and lipids, thereby generating diverse damage responses. OXPHOS represents the major site of ROS generation since it represents nearly 85% to 90% of the cell oxygen consumption (Kwon et al., 2019). Current data showed that basal mitochondrial respiration did not change over time in *E. bovis*–infected cells but intracellular ROS production was indeed enhanced during macromeront development. It is worth noting that OXPHOS-related proteins are coded in the mitochondrial DNA (mtDNA), which is highly vulnerable to contact with ROS molecules due to its localizationclose to ROS site production in the mitochondria, which suggests that *E. bovis* infection may not only affect nuclear DNA but mtDNA as well. Besides, mitochondrial ROS may activate an adaptative cellular response called mitohormesis, defined as a mitochondrial defensive mechanism that extends the cellular lifespan and delays cell death (Ristow and Schmeisser, 2011; Scheibye-Knudsen et al., 2015; Davalli et al., 2016). Therefore, mitohormesis could be beneficial for appropriate *E. bovis* macromeront long-term development with massive parasite replication. In this scenario, the NADPH molecule can protect against oxidative stress directly by neutralizing reactive oxygen intermediates or indirectly *via* regenerating reduced glutathione (GSH) from its oxidized form GSSG (Fernandez-Marcos and Nóbrega-Pereira, 2016). Besides, the glutathione peroxidase-dependent reduction of ROS requires a continuous supply of NADPH for regeneration (Aon et al., 2012). Our current data revealed a parasite-triggered enhancement of NADPH production exclusively at 22 days p. i., which matches with the higher peak of intracellular ROS. Probably, at this point of the infection, the ROS concentrations reach toxic levels for the host cell, which activates the NADPH production as well as the glutathione reductase pathway to protect the host cell from oxidative stress damage. Another possible intracellular fate of NADPH is (turning it into anabolic reactions, acting as the main reductant). NADPH participates in the fatty acids and some amino acids biosynthesis, while NADP is necessary to produce triacylglycerols, phospholipids, and steroids, such as cholesterol, bile acids, and steroid hormones (Agledal et al., 2010). Overall, the current data indicate that *E. bovis* not only induces glycolysis for its benefit but also modulates detoxification mechanisms and mitochondrial function in primary endothelial host cells.

## Data Availability Statement

The original contributions presented in the study are included in the article/supplementary material. Further inquiries can be directed to the corresponding author.

## Author Contributions

ZV and AT: designed the project and experiments. ZV and SL-O: carried out the experiments. ZV: Images, video acquisition, and image analysis. ZV: Figure’s preparation. SM: scheme design. ZV, SM, CH, and AT: Prepared the manuscript. All authors reviewed the manuscript. All authors contributed to the article and approved the submitted version.

## Conflict of Interest

The authors declare that the research was conducted in the absence of any commercial or financial relationships that could be construed as a potential conflict of interest.
